# Dual-Responsive Hydrogels for Mercury Ion Detection and Removal from Wastewater

**DOI:** 10.3390/gels10020113

**Published:** 2024-02-01

**Authors:** Aurel Diacon, Florin Albota, Alexandra Mocanu, Oana Brincoveanu, Alice Ionela Podaru, Traian Rotariu, Ahmad A. Ahmad, Edina Rusen, Gabriela Toader

**Affiliations:** 1 Military Technical Academy “Ferdinand I”, 39-49 G. Cosbuc Blvd., 050141 Bucharest, Romania; aurel_diacon@yahoo.com (A.D.); podaru.alice04@gmail.com (A.I.P.); traian.rotariu@mta.ro (T.R.); 2 Faculty of Chemical Engineering and Biotechnologies, National University of Science and Technology Politechnica Bucharest, 1-7 Gh. Polizu Street, 011061 Bucharest, Romania; alexandra.mocanu@imt.ro; 3 Horia Hulubei National Institute of Physics and Nuclear Engineering, 30 Reactorului Street, 077125 Magurele, Romania; florin.albota@nipne.ro; 4 National Institute for Research and Development in Microtechnologies—IMT Bucharest, 126A Erou Iancu Nicolae Street, 077190 Bucharest, Romania; oana.brincoveanu@imt.ro; 5 Research Institute, University of Bucharest, 90 Sos. Panduri, 050663 Bucharest, Romania; 6 Department of Physical Sciences, Jordan University of Science & Technology, P.O. Box 3030, Irbid 22110, Jordan; sema@just.edu.jo

**Keywords:** hydrogels, water treatment, mercury, rhodamine, phytic acid, IPN, decontamination

## Abstract

This study describes the development of a fast and cost-effective method for the detection and removal of Hg^2+^ ions from aqueous media, consisting of hydrogels incorporating chelating agents and a rhodamine derivative (to afford a qualitative evaluation of the heavy metal entrapment inside the 3D polymeric matrix). These hydrogels, designed for the simultaneous detection and entrapment of mercury, were obtained through the photopolymerization of 2-acrylamido-2-methyl-1-propanesulfonic acid (AMPSA) and N-vinyl-2-pyrrolidone (NVP), utilizing N,N′-methylenebisacrylamide (MBA) as crosslinker, in the presence of polyvinyl alcohol (PVA), a rhodamine B derivative, and one of the following chelating agents: phytic acid, 1,3-diamino-2-hydroxypropane-tetraacetic acid, triethylenetetramine-hexaacetic acid, or ethylenediaminetetraacetic acid disodium salt. The rhodamine derivative had a dual purpose in this study: firstly, it was incorporated into the hydrogel to allow the qualitative evaluation of mercury entrapment through its fluorogenic switch-off abilities when sensing Hg^2+^ ions; secondly, it was used to quantitatively evaluate the level of residual mercury from the decontaminated aqueous solutions, via the UV-Vis technique. The ICP-MS analysis of the hydrogels also confirmed the successful entrapment of mercury inside the hydrogels and a good correlation with the UV-Vis method.

## 1. Introduction

Mercury contamination of land, air, and water poses a serious threat to public health and the environment. Wastewater treatment is essential for reducing the amount of mercury released into rivers and lakes. As strong adsorbent materials, hydrogels are a feasible option to remove hazardous heavy metals from wastewater. Mercury is the third most common and second most hazardous heavy metal, according to the Agency for Toxic Substances and Disease Registry (ATSDR) from the U.S. Department of Health and Human Services [1,2]. As a result, monitoring the presence of Hg^2+^ is critical. Depending on the application, fluorescence sensing could represent a rapid and feasible method to detect Hg^2+^ ions with high sensitivity and efficiency. Qualitative methods can provide an initial estimate of mercury contamination, but a quantitative method is always necessary for precise evaluation. Atomic absorption spectroscopy (AAS) and inductively coupled plasma mass spectrometry (ICP-MS) are highly accurate techniques for the quantification of mercury contamination levels, but their main disadvantage is related to the expensive cost considerations. Ultraviolet–visible (UV–Vis) or fluorescence spectroscopy may sometimes be useful to evaluate mercury levels, but a model dye is necessary. In contrast to AAS and ICP-MS, UV-Vis or fluorescence spectroscopy could represent more straightforward methods for mercury quantification, due to their higher availability, lower costs, and ease of application, but to some extent lower accuracies.

Several treatment technologies have been intensively researched to remove metal ions from wastewater, including ion exchange, reverse osmosis, adsorption, and precipitation [3]. Adsorption is typically chosen over the other techniques because of its superior efficiency, simplicity in use, and diversity of readily accessible adsorbents [3].

Due to their swelling response to ionic strength, pH, or temperature, numerous studies focused on the utilization of hydrogels with amide, amine, carboxylic acid, or ammonium groups, as adsorbents that can bind heavy metal ions to remove them from aqueous environments [3,4]. The most commonly used synthetic monomers for hydrogel synthesis are acrylic acid (AA), methacrylic acid (MAA), itaconic acid (IA), maleic acid (MA), 2-acrylamide-2-methylpropane sulfonic acid (AMPSA), acrylamide (AM), N-isopropyl acrylamide (NIPAM), N-vinyl-2-pyrrolidone (NVP), methyl methacrylate (MMA), hydroxyethyl methacrylate (HEMA), ethylene glycol dimethacrylate (EGDMA), etc. [5]. Due to their high absorption capacity when swelled in water, hydrogels based on 2-acrylamido-2-methyl-1-propanesulfonic acid (AMPS) [6] have gained attention in water treatment applications. Nevertheless, poly(AMPSA) hydrogels have reported poor mechanical characteristics, probably due to their covalently cross-linked network structure, which prevents the crack energy from dissipating [6]; consequently, to obtain stronger PAMPSA-based hydrogels, which preserve their integrity and mechanical resistance even at high swelling ratios, other distinct monomeric units can be incorporated into the crosslinked network without compromising their superabsorbency [6,7]. N-vinylpyrrolidone (NVP) is one of the co-monomers successfully utilized in AMPSA-based hydrogels due to its hydrophilic nature and the biocompatibility of poly(NVP) [8,9].

Hydrogels may easily swell in an aqueous environment and can be designed to sensibly respond to variations in external stimuli, due to their versatility and their three-dimensional hydrophilic polymeric networks [10,11,12]. Based on their shape, the hydrogels that are frequently used in water and wastewater treatment can be divided into three classes: hydrogel films, hydrogel beads, and hydrogel nanocomposites [4]. Regardless of their shape, integrating an interpenetrating polymeric network (IPN) inside the hydrogels designed for water treatment applications may prove to be advantageous, since high swelling levels occasionally result in poor mechanical resistance. The crosslinked components of an IPN hydrogel have been hypothesized to preserve the stability associated with the bulk and surface morphology of the hydrogels because they possess interconnected networks that typically consist of two or more polymeric chains, with at least one polymerized and/or crosslinked in the immediate presence of others [3]. Polyvinyl alcohol (PVA) is a water-soluble synthetic polymer that is widely utilized in the production of IPN hydrogels because it enhances their mechanical and physical properties, making them more stable and resilient [13,14,15].

Hydrogels have recently been studied for the removal of various metal ions [16,17,18,19,20,21] and also for their optical detection. To enable hydrogels for contaminant sensing applications, stimuli-responsive molecules are often incorporated into the structure of their crosslinked polymeric network. Liu et al. developed a portable hydrogel sensor for Fe^3+^ based on a rhodamine derivative [22]. A rhodamine-immobilized optical hydrogel for the selective detection of Hg^2+^ was recently reported by Qu et al. [8]. Rhodamine-based colorimetric or fluorometric Hg^2+^ probes have been obtained using chromophore/fluorophore units attached to synthetic polymer [23,24], natural polymer [25], silica [26,27], or zeolites [28]. Other fluorescent sensors reported for Hg^2+^ include the following examples: 1,8-naphthalimide [2], fluorescein [29], BODIPY [30], etc. According to Li Qiuping et al. [31], fluorescent sensors frequently utilize multiple sensing mechanisms of Hg^2+^, and their main sensing mechanisms are intramolecular charge transfer, ligand-to-metal energy transfer, static quenching, photoinduced electron transfer, mercury-induced reaction, metallophilic interaction, and aggregation-induced emission. Due to their excellent selectivity, high sensitivity, low cost, accessibility, and suitability for on-site and real-time signaling, fluorescent sensors have become highly sought after in the field of heavy metal ion detection [1]. The benefits of rhodamine dyes include broad excitation and emission wavelengths, durable photostability, easily modifiable basic frameworks, and remarkably vivid color shifts when the analyte is present [1,2,10,32]. Several studies revealed a high selectivity of rhodamine derivatives for mercury ions [2,16,33]. Although rhodamine-based fluorescent sensors for Hg^2+^ have been the subject of numerous studies [33], there is still an opportunity to further enhance the development of these rhodamine-based sensing hydrogels by designing easier fabrication procedures, which ensure higher sensitivity, good selectivity, and fast reaction times.

In the actual context, the aim of this study was to develop hydrogels incorporating a rhodamine derivative and four distinct types of chelating agents for the removal of mercury ions from aqueous media. The role of the chelating agents inside the 3D polymeric network is to capture mercury ions, while the role of the rhodamine derivative is to confirm the entrapment of mercury via its fluorogenic switch-off response when sensing the Hg^2+^ ions. The synthesized rhodamine derivative served a dual purpose: it was incorporated into the hydrogel to afford an optical, qualitative monitorization of the heavy metal entrapment and it was also utilized for both qualitative and quantitative monitorization of the mercury removed from the decontaminated aqueous solutions, via UV-Vis. ICP-MS analysis also confirmed the successful entrapment of mercury inside the IPN hydrogels.

The novelty of this paper resides in the versatility of employing the rhodamine derivative (for sensing Hg levels and decontamination efficacy) inside the IPN hydrogels (poly(NVP-co-AMPSA)) and their remarkable decontaminating capacity obtained by incorporating the chelating agents in the 3D polymeric matrix (phytic acid, 1,3-diamino-2-hydroxypropane-N,N,N′,N′-tetraacetic acid, triethylenetetramine-N,N,N′,N″,N‴,N″″-hexaacetic acid, and ethylenediaminetetraacetic acid disodium salt), empowering these IPN hydrogels with synergistic sensing and decontaminating abilities. Accordingly, the hydrogels developed within this research study could be effectively applied in water treatment applications for heavy metal decontamination, while the rhodamine derivative facilitates the monitorization of the Hg^2+^ removal efficiency in aqueous media.

## 2. Results and Discussion

### 2.1. Method Principles

The present study presents an innovative method for the detection and removal of Hg^2+^ ions from aqueous media. The approach involves the development of a rapid and cost-effective technique that utilizes hydrogels in combination with chelating agents to entrap the contaminants. Additionally, a rhodamine B derivative is used to qualitatively evaluate the heavy metal entrapment inside the 3D polymeric matrix. This innovative technique has the potential to provide an efficient and sustainable solution for removing heavy metals from water, which is of great significance in various industrial and environmental applications.

**Figure 1:**
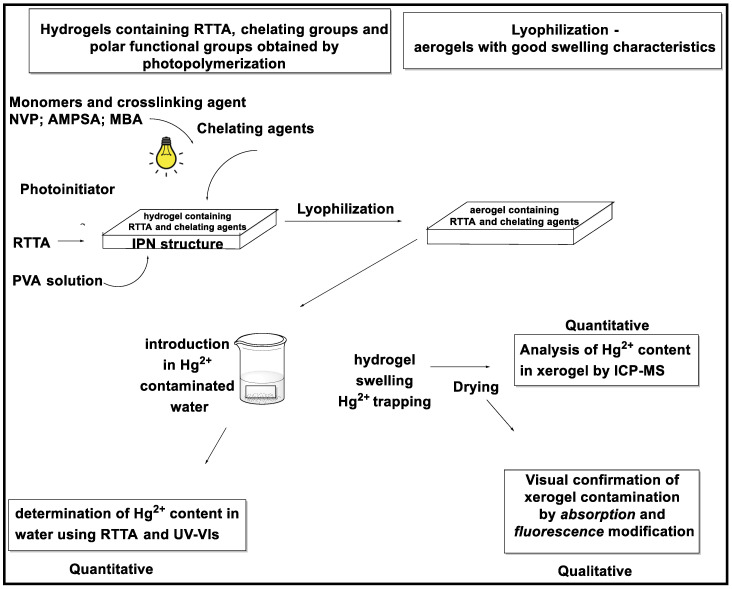
Method principles.

Figure 1 schematically illustrates the principles of this decontamination method implying the poly(NVP-co-AMPSA)-based hydrogels, incorporating chelating agents and a rhodamine derivative, designed for Hg^2+^ detection and removal from wastewater.

The principle of this method implies allowing the aerogel (incorporating the rhodamine derivative RTTA and phytic acid as a chelating agent in this example) to swell in the mercury-contaminated aqueous solution, and to absorb and entrap the Hg^2+^ ions in its 3D polymeric network. In the presence of the heavy metal contaminant, a fluorogenic switch-off sensing of Hg^2+^ occurs for RTTA, thus allowing a qualitative evaluation of the decontamination progress.

### 2.2. Morphological Characterization of the Hydrogels

**Figure 2:**
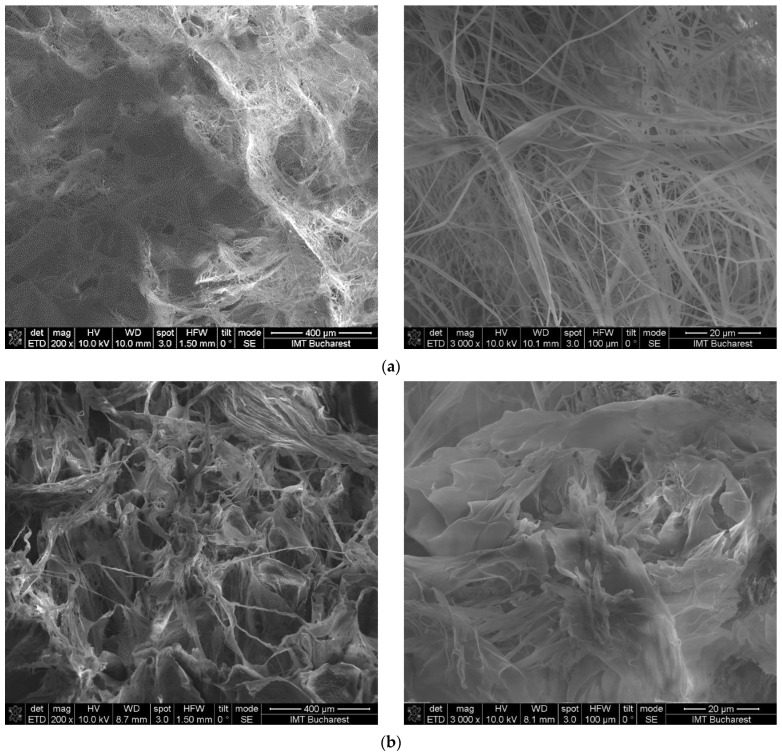
SEM images of the lyophilized hydrogels: (**a**) blank sample (left: 400 µm scale; right: 20 µm scale); (**b**) sample containing phytic acid (left: 400 µm scale; right: 20 µm scale).

SEM analysis (Figure 2 and Figure S1) revealed the macroporous morphology of the lyophilized hydrogels. The blank sample (Figure 2a), containing PVA chains interpenetrated with poly(NVP-co-AMPSA) units crosslinked with MBA, displayed a soft spongious morphology comprised of thin fibrillar structures.

As can be observed from Figure 2b and Figure S1a–c, the other four types of hydrogels, which contain chelating agents, possess a morphology comprised of interconnected polyhedral [34] cavernous pores, with more continuous pore walls than the blank sample, probably due to a denser network created through the supplementary intermolecular interactions (hydrogen bonding) induced by the presence of the chelating agents inside the polymeric network [6,35] and the additional ionic interactions established by the sulfonate groups from AMPSA or by the NVP moieties with the chelates [35,36,37,38]. The structure of these lyophilized hydrogels may be explained by the effect induced by the freezing of free water inside the polymer network meshes, which may lead to the deformation of the pores, by stretching the hydrated chains around the ice crystals formed [34].

### 2.3. Gel Fraction and Swelling Degree

**Figure 3:**
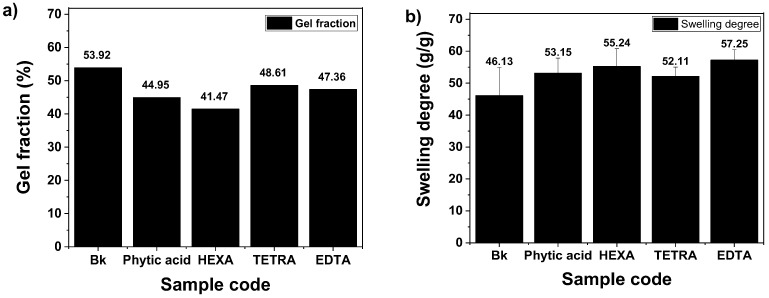
Gel fraction and swelling degree obtained for the synthesized hydrogels: (**a**) Gel fraction; (**b**) Swelling degree.

The five types of hydrogels synthesized for this study (monomer conversions ≥98%, in all cases) were analyzed to determine the gel fraction (Figure 3a) and their ability to absorb water (Figure 3b). The cross-linking efficiency between the polymeric chains inside a hydrogel can be evaluated through the gel fraction [39,40]. Figure 3a shows that the hydrogels exhibited relatively close gel fraction values. The ability of the hydrogels to swell decreases with the increase in cross-linking density [39], which is confirmed by the swelling capacity of the Bk sample compared with the rest. The polymerization reaction rate and, consequently, the crosslinking degree are dependent on the concentration of free monomer (NVP) in the system [41,42]. Thus, in the case of TETRA and EDTA, which present four carboxyl groups, a higher crosslinking degree was noticed compared with HEXA and PHYTIC, which displayed lower gel fractions, each presenting six acidic functional groups. Thus, the higher swelling capacities of the hydrogels containing chelating agents are explainable by the lower crosslinking density but also the values can be explained by the presence of supplementary ionic moieties [43] and by a higher pliability [43] of the IPN, which allow a higher intake of water. Nevertheless, according to the data obtained, we can affirm that the presence of these chelating agents during the photopolymerization process does not decrease the swelling properties of these hydrogels intended for water treatment applications.

### 2.4. FT-IR Analysis

**Figure 4:**
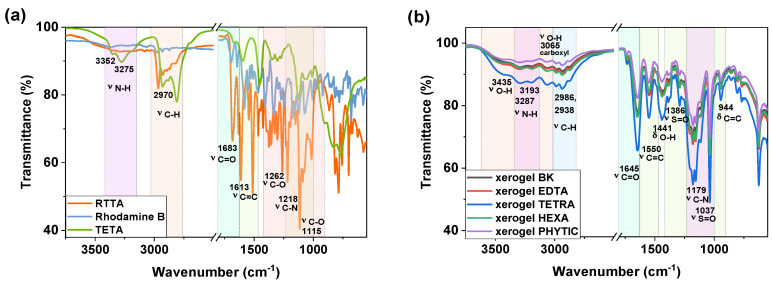
FTIR comparative multigraphs for (**a**) rhodamine B, RTTA, and TETA; (**b**) aerogels.

FT-IR analysis revealed the characteristic vibrational bands for RTTA (Figure 4a) and for the synthesized hydrogels (Figure 4b and Figure S2). In Figure 4a, due to the functionalization of the RB molecule with TETA, the RTTA sample showed the growth of these distinct peaks for the primary and secondary amines, with the specific vibrational bands νNH~3400 cm^−1^ and 3300 cm^−1^, respectively. As can be observed from Figure 4b and Figure S2, the carbonyl group N–C=O specific for NVP moiety is noticeable at 1645 cm^−1^, while the absorption bands around 1387 cm^−1^ could be attributed to the characteristic S=O stretching from AMPSA. The other minor differences that appear between the FT-IR plots are due to the chelating agents incorporated into the hydrogels.

### 2.5. Mechanical Properties of the Hydrogels

**Figure 5:**
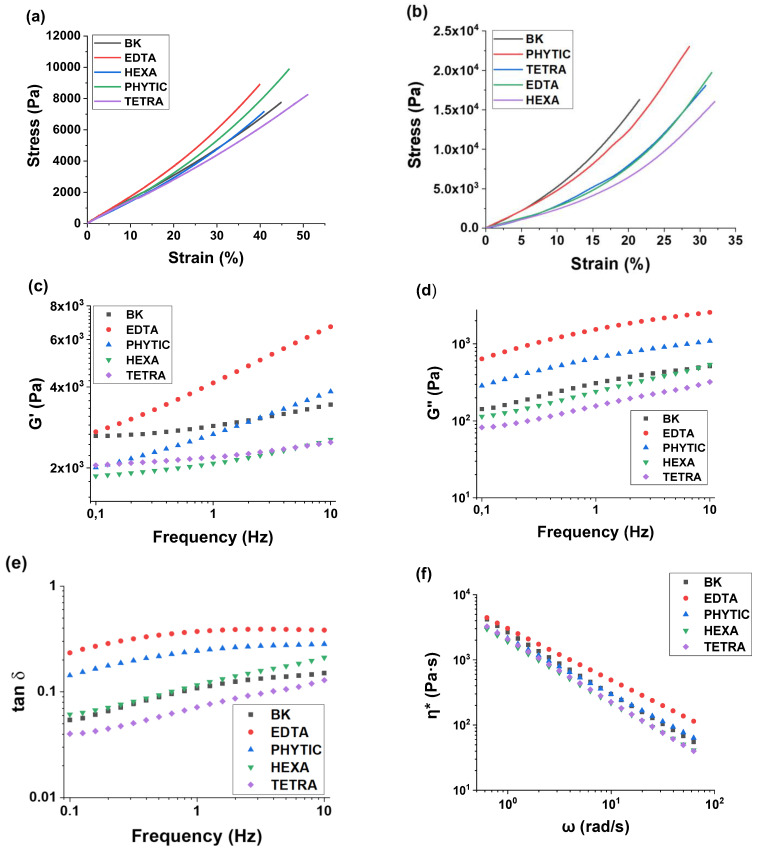
Mechanical properties of the hydrogels: (**a**) tensile loading mode; (**b**) compression loading mode; (**c**,**d**) shear loading mode; (**e**) tan δ multigraph; and (**f**) complex viscosity η vs. angular frequency.

Ensuring adequate mechanical resistance in hydrogels designed for water treatment is crucial to avoid the risk of fracture and facilitate their recovery after the decontamination process. Thus, the mechanical properties of the synthesized hydrogels were further evaluated, and the results obtained are displayed in Figure 5. The synthesized hydrogels were first subjected to tensile and compression tests to evaluate their mechanical resistance under tensile or compressive loads. Next, the viscoelastic properties of the hydrogels were evaluated because they can also have a significant impact on the decontamination process.

The tensile test (Figure 5a) revealed the influence of the chelating agents on the mechanical resistance of the hydrogels. Except the sample containing 1,3-diamino-2-hydroxypropane-N,N,N′,N′-tetraacetic acid (TETRA), all the other hydrogels incorporated chelating agents displayed a slightly higher mechanical resistance than the blank sample. The slight differences between the tensile resistance of the hydrogels containing chelating agents may appear due to the distinct ionic interactions established with the NVP moieties from the IPN. The sample containing phytic acid led to the highest ultimate stress values, probably due to the significant strengthening effect [6,44] of supplementary H-bonding interactions established with the NVP and AMPSA moieties. The amount of chelating groups, thus the supplementary interactions established, influence the density of the network and, thus, the mechanical resistance of the hydrogels. The compression tests (Figure 5b), performed on samples in their equilibrium swollen state, revealed lower stress values and higher strain values for the hydrogels containing chelating agents. This behavior can be explained by their anisotropy [43] but also through their higher swelling degrees, which imply a higher water content and a lower stiffness [45]. The viscoelasticity of hydrogels can be correlated with their microstructures, which may offer valuable insights for adjusting their performance characteristics. The viscoelastic properties of hydrogels depend on their composition but also on the interactions established with the liquid confined in their 3D polymeric network. The storage and loss moduli (Figure 5c,d) describe the viscoelastic properties of the synthesized hydrogel films. All samples revealed that storage moduli (G′) values were greater than (G″) values, which is a common characteristic of crosslinked hydrogels [46]. A higher storage modulus (G′) indicates that elastic behavior becomes dominant, while the influence of the viscous behavior is diminished [47], as frequency increases. An important technique employed in the characterization of hydrogels is small-amplitude oscillatory shear (SAOS) [48]. Important details about gel structure and mechanical behavior can be deduced from the frequency dependence of the dynamic moduli G′ and G″ in the linear-viscoelastic regime (LVE) [48]. Since rheometers have limited sensitivity, choosing the applied strain amplitude requires finding a balance between the requirement to preserve sample integrity and the necessity to give a sufficiently strong signal. Thus, the hydrogels reported herein were analyzed in frequency sweep mode, on a frequency range between 0.1–10 Hz, at a constant oscillation strain of 10%. Tan delta (δ) shows the ratio of the viscous to elastic effects and reveals how the sample transitions from solid- to liquid-like behavior as a function of the experimental timescale (Figure 5e). When tan δ > 1, it indicates that G″ has a superior value than G′ and that the material is more viscous, allowing for greater energy dissipation; however, when tan delta < 1, it indicates that G′ has a greater value than G″ and that the material is elastic [49]. Thus, the tan delta plot also confirms the elastic behavior tendency of the herein-reported crosslinked hydrogels. However, samples with greater tan δ values tend to have a more viscous character. Complex viscosity measurements [23] (Figure 5f) can be associated with hydrogel structural strength, and they revealed that all hydrogels possess pseudoplastic properties [50]. The increase in the shear rate leads to a deformation of the polymeric chain entanglements as a result of the breakdown of physical interactions, resulting in a decrease in the viscosity values.

### 2.6. Hg^2+^Decontamination Survey

To evaluate their ability to entrap, detect, and remove the Hg^2+^ ions from an aqueous solution, the aerogels (containing RTTA and a chelating agent) were immersed in a HgCl_2_ aqueous solution. After 48 h, they were removed from the decontaminated aqueous solution and allowed to dry.

**Figure 6:**
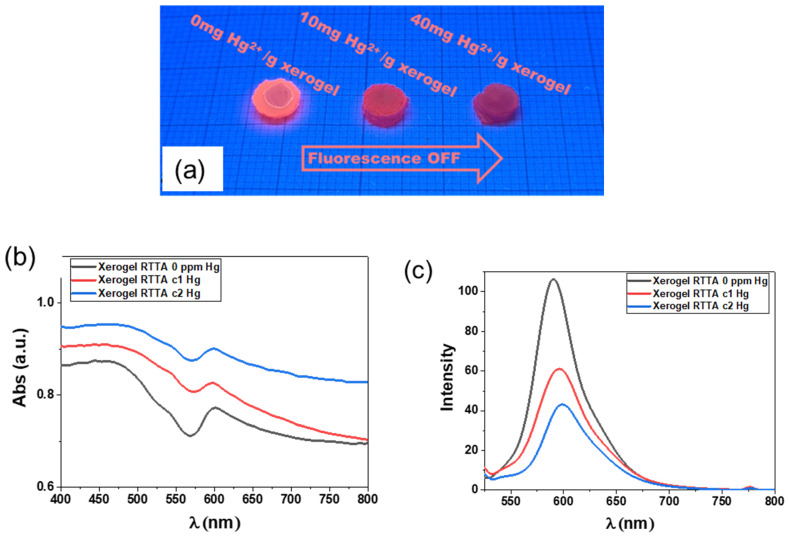
(**a**) Images of fluorescence modification with the increase in the amount of Hg^2+^ absorbed; (**b**) UV-Vis spectra of the aerogel samples with different Hg^2+^ concentrations; and (**c**) emission spectra of the aerogel samples with different Hg^2+^ concentrations, excitation wavelength 520 nm.

**Figure 7:**
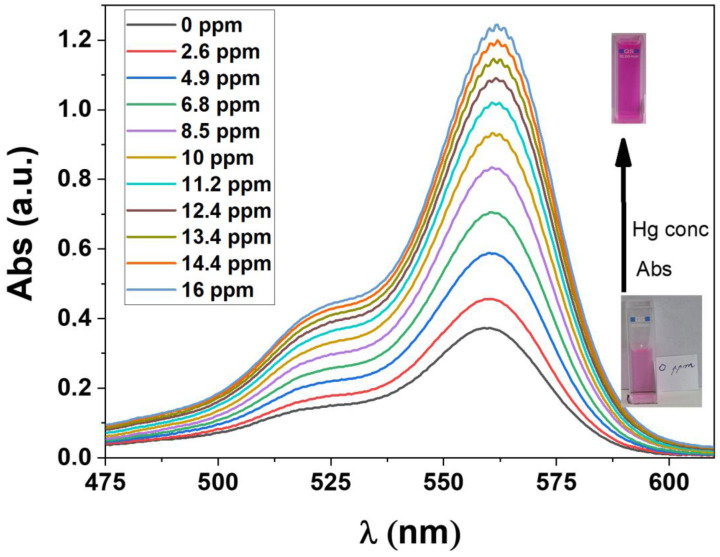
UV-Vis survey: RTTA absorbance vs. Hg^2+^ conc.

**Figure 8:**
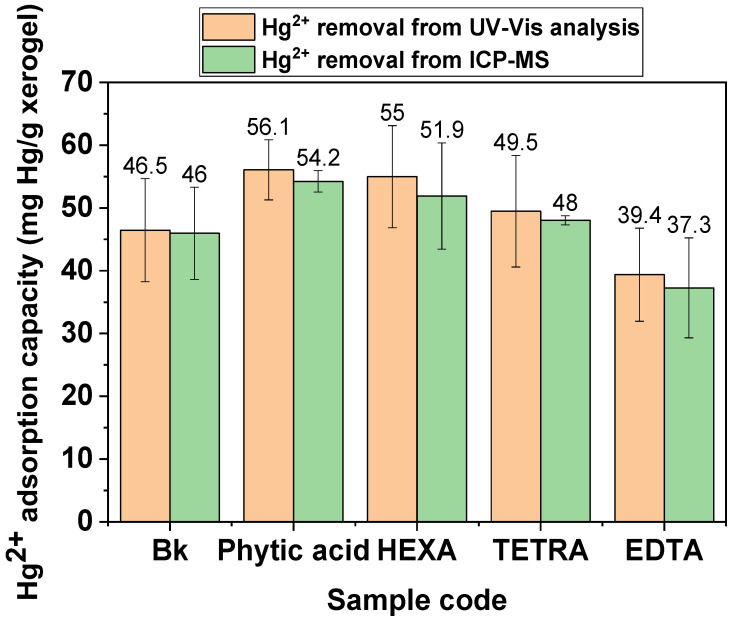
Comparative plots for the efficacy of mercury removal via UV-Vis and ICP-MS.

As can be observed from Figure 6, this method allows a fast, efficient route for confirming the presence of Hg^2+^, but could also allow a qualitative evaluation of the mercury levels. Initially, at low Hg^2+^ concentrations, the fluorescence is increased due to a fluorescence “on” effect (Scheme S1); however, at higher Hg^2+^ concentrations and due to the high RTTA amount in the aerogels, the fluorescence is gradually turned off [51,52]. The fluorogenic switch-off (Scheme S2) sensing of Hg^2+^ [31] could be explained by the interruption of the intramolecular conjugation in RTTA molecules through the new interactions established by the nitrogen atoms with Hg^2+^ ions. For a more accurate evaluation of the mercury removal efficacy, quantitative methods were employed, which are further described. The mercury decontamination survey was performed following two routes: (i) UV-Vis analysis (Figure 7 and Figure S3) of the decontaminated aqueous solutions (post-decontamination residual mercury), and (ii) ICP-MS analysis of the absorbent (mercury entrapped in the aerogels). The results obtained are illustrated comparatively in Figure 8. A good correlation between the results was obtained from the two analytical techniques employed in the Hg^2+^ decontamination survey. The samples incorporating phytic acid revealed the best removal efficacy for mercury, followed by the ones containing triethylenetetramine-hexaacetic acid (HEXA) and 1,3-diamino-2-hydroxypropane-tetraacetic acid (TETRA), while the samples containing EDTA led to inferior results. The results obtained were in accordance with the amount of chelating units incorporated into the hydrogels and are dependent on the molecular structures of the Hg^2+^/chelating agent complexes formed [53,54,55].

The characteristics of the UV-Vis method are presented comparatively in Table 1. As can be noticed, the RTTA derivative displays a relatively low detection limit, which is interesting since it involves a colorimetric method. Lower detection limits can be achieved via fluorescence modification evaluation. Furthermore, the interaction with other metal cations requires attention (Figures S4 and S5).

## 3. Conclusions

In this study, we synthesized five types of hydrogels, via photopolymerization, for the detection and removal of mercury ions from wastewater. The morphology of these hydrogels was evaluated with SEM, indicating that the samples containing chelating agents possess polyhedral cavernous pores. FT-IR analyses indicated the characteristic vibrational bands for the synthesized rhodamine derivative (RTTA) and for the hydrogels containing RTTA and the four types of chelating agents. The hydrogels were subjected to mechanical investigations, which included tensile and compression tests to measure their resistance to such loads. Additionally, the viscoelastic properties were evaluated, as they can play a crucial role in the decontamination process. Based on the experimental results, it appears that phytic acid has had a notable positive impact on the mechanical strength of the hydrogels when compared with the other chelating agents tested. When performing the decontamination survey, it was noticed that the fluorescence of the post-decontamination aerogels was gradually turned off with the presence of higher concentrations of Hg^2+^ ions. UV-Vis and ICP-MS showed a good correlation between the results obtained from the Hg^2+^ decontamination survey. Based on the amount of chelating units incorporated into the hydrogel, the samples that contained phytic acid exhibited the highest efficacy in removing mercury, followed by those containing triethylenetetramine-hexaacetic acid (HEXA) and 1,3-diamino-2-hydroxypropane-tetraacetic acid (TETRA). However, the samples containing EDTA did not yield the desired results. In contrast, the samples containing phytic acid were capable of entrapping up to 56 mg of Hg^2+^ per gram of aerogel.

This research study has developed promising hydrogels that can be utilized for the efficient removal of heavy metals from water. The use of the rhodamine derivative also allows the convenient determination of Hg^2+^ removal efficacy in aqueous environments via the UV-Vis method. These findings offer potential solutions for water treatment applications and may contribute to the development of safer and cleaner water resources.

## 4. Materials and Methods

### 4.1. Materials

Poly(vinyl alcohol) (PVA, average Mw 85,000–124,000 Da, 99%+ hydrolysis degree, Sigma Aldrich, Saint Louis, MO, USA), 2-Acrylamido-2-methyl-1-propanesulfonic acid (AMPSA, 99%, Sigma Aldrich, Saint Louis, MO, USA), N,N′-methylenebisacrylamide (MBA, 99%, Sigma Aldrich, Saint Louis, MO, USA), 2-hydroxy-4′-(2-hydroxyethoxy)-2-methylpropiophenone (Ph-In, 98%, Sigma-Aldrich, Saint Louis, MO, USA), rhodamine B (RB, analytical standard, Sigma-Aldrich, Saint Louis, MO, USA), triethylenetetramine (TETA, Sigma-Aldrich, Saint Louis, MO, USA), ethanol (EtOH, Sigma-Aldrich, Saint Louis, MO, USA), dichloromethane (DCM, ≥99.8%, Sigma-Aldrich, Saint Louis, MO, USA), phytic acid (Phytic acid, sodium salt hydrate from rice, Sigma-Aldrich, Saint Louis, MO, USA), 1,3-diamino-2-hydroxypropane-N,N,N′,N′-tetraacetic acid (TETRA, 99%, Sigma-Aldrich, Saint Louis, MO, USA), triethylenetetramine-N,N,N′,N″,N‴,N″″-hexaacetic acid (HEXA, ≥98.0%, Sigma-Aldrich, Saint Louis, MO, USA), ethylenediaminetetraacetic acid (EDTA, ethylenediaminetetraacetic acid disodium salt dihydrate, 99.0%, Sigma-Aldrich, Saint Louis, MO, USA), mercury(II) chloride (HgCl_2_, 99%, Sigma-Aldrich, Saint Louis, MO, USA), and deionized water were used as received. 1-vinyl-2-pyrrolidone (NVP, sodium hydroxide inhibitor, ≥99%, Sigma Aldrich, Saint Louis, MO, USA) was distilled and stored at 4 °C before the synthesis steps.

### 4.2. Methods

#### 4.2.1. Synthesis of Rhodamine-TETA Derivative (RTTA)

**Scheme 1:**
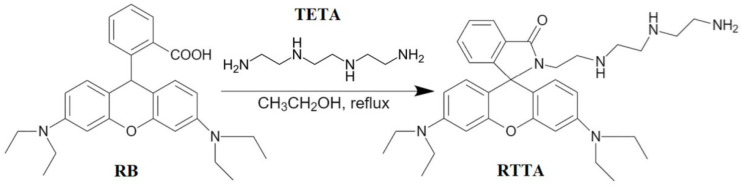
Rhodamine-TETA (RTTA) derivative synthesis.

Scheme 1 illustrates the synthesis route of the rhodamine derivative (RTTA), which was obtained according to Refs. [33,62,63].

For the synthesis of RTTA, 0.4 g of RB was dissolved in 40 mL of ethanol, followed by the dropwise addition of 4 mL of TETA, under vigorous stirring. After heating the mixture to reflux for 8 h, its color changed from red to pale yellow. A low-pressure rotary evaporator was used to extract the solvent. After adding water to the solid residue, dichloromethane was used to extract RTTA. The organic phase was washed three times with water, followed by solvent evaporation. The RTTA was vacuum-dried, yielding a yellowish solid (90%).

#### 4.2.2. Synthesis of RTTA-Based Hydrogels Incorporating Distinct Chelating Agents

**Scheme 2:**
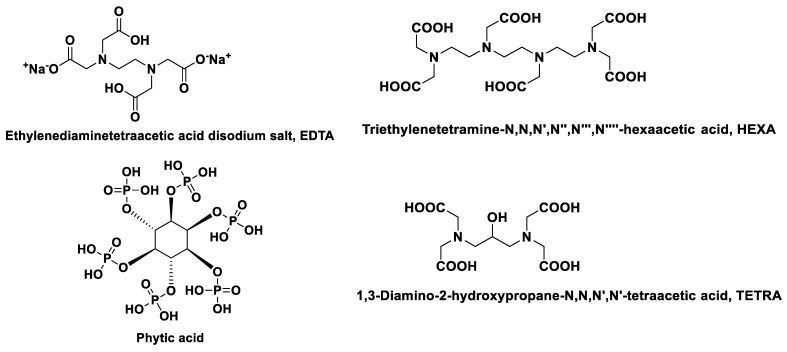
Chelating agents incorporated into the hydrogel films.

For the blank sample (Bk), the monomers (3.33 g NVP and 1.725 g AMPSA), the crosslinker (0.1 g MBA), 0.005 g RTTA, and the photoinitiator (0.028 g Ph-In) were dissolved in 10 g of PVA aqueous solution (5 wt.%). In addition, for the hydrogels incorporating chelating agents, the appropriate amounts (Table 2) of EDTA, HEXA, TETRA, or phytic acid (Scheme 2) were dissolved in the PVA aqueous solution (5 wt.%). The formulations described in Table 2 were utilized to obtain five distinct types of hydrogel films via photopolymerization, using a UV lamp (low-pressure Hg UV lamp, λem = 254 nm), employing circular (silicone rubber-sealed) glass molds (⌀ 5 cm × 0.2 cm). After approximately 40 min of UV exposure, the IPN films were completely cured.

#### 4.2.3. Characterization

SEM imaging of the lyophilized hydrogels was performed at 10 kV using a field emission gun scanning electron microscope (FEGSEM), Nova NanoSEM 630 (FEI) (Hillsboro, OR, USA). The FT-IR analysis of RB, TETA, RTTA, the five aerogels, and the chelating agents was performed on a Spectrum Two FTIR spectrometer (PerkinElmer, Waltham, Massachusetts, United States) equipped with a MIRacleTM Single Reflection ATR (PIKE Technologies), at 4 cm^−1^ resolution, totaling 32 scans, and 4000–600 cm^−1^ wavenumber range. For estimating the gel fraction [39], the hydrogels were dried at 60 °C, in an oven, to constant weight (*w*_0_). They were subsequently immersed in distilled water. After being maintained for 24 h in distilled water, filter paper was used to wipe the hydrogels, and they were weighed (*w*_1_). The gel fraction was calculated according to Equation (1):

(1) $$Gel\;fraction\;\left(\%\right)=\frac{w_{1}}{w_{0}}\times 100$$

where *w*_1_ (*g*) is the weight of the hydrogel after being immersed in distilled water for 24 h, and *w*_0_ (*g*) is the weight of the dry hydrogel before immersion in distilled water. The swelling ability of the hydrogels (Equation (2)) was assessed according to Refs. [64,65,66]. Aerogels (*w_x_*) were weighted and then further immersed and maintained (at 37 °C) in distilled water until they reached a constant weight (*w_h_*):

(2) $$Swelling\;degree\;\left(g/g\right)=\frac{w_{h}-w_{x}}{w_{x}}$$

where *w_h_* (*g*) represents the weight of the hydrogel in the equilibrium swollen state, and *w_x_* (*g*) is the weight of the completely dried aerogel. The mechanical properties of the synthesized hydrogels were evaluated using a DMA 850 instrument from TA Instruments, utilizing specific clamps for each type of test, as follows: the tensile clamp was utilized for subjecting five rectangular hydrogel specimens from each type of sample to the uniaxial tensile test, performed at 5 mm/min in rate control–strain ramp mode, and the mean values were reported; the compression clamps (Ø40 mm) were utilized for subjecting five fully swollen disc specimens from each sample to uniaxial compressive loading at 2 mm/min, in rate control–strain ramp mode, and mean values were reported; and the "shear-sandwich" clamps were employed to evaluate the frequency-dependent shear modulus, in oscillation–frequency sweep mode, at a constant strain of 10%, with a logarithmic increase in the frequency from 0.1 to 10 Hz.

To evaluate the Hg^2+^ decontamination performances of the herein-reported hydrogels, UV-Vis and ICP-MS techniques were employed. Prior to UV-Vis and ICP-MS evaluation, the aerogels were immersed in a 1000 ppm HgCl_2_ solution and maintained at 20 °C under orbital stirring in a dry bath (DLAB HCM100-Pro, Riverside, CA, USA). After 48 h, the hydrogels were removed from the aqueous HgCl_2_ solutions. The remaining aqueous solutions were analyzed with the aid of RTTA via UV-VIS, while the hydrogels were completely dried in an oven and further subjected to ICP-MS analysis. These two types of assessments are described in detail below:

For the UV-VIS assessment of the removal efficacy, the Hg^2+^ levels from the HgCl_2_ aqueous solutions were estimated according to the absorbance changes in RTTA, using the calibration curve presented in the Supplementary Materials Section. Thus, an RTTA solution, c_M_ = 4.5 × 10^−4^ M (maximum absorption wavelength λ = 560 nm), ethanol: deionized water 1:1 (*v*/*v*) as the solvent, served for the spectrophotometric evaluation of Hg^2+^ in the decontaminated solutions. For this purpose, a UV-Vis Cintral303 Spectrophotometer instrument, provided with a double beam in the photometric range 190–900 nm, was utilized to quantify the absorbance modifications of RTTA solutions in the presence of Hg^2+^ ions. All absorbance measurements were taken in 10 mm width UV-Vis quartz spectroscopy cells, at room temperature. For the UV-Vis measurements, 0.2 mL of the RTTA solution was mixed with 1 mL of deionized water, 1 mL of ethanol, and 0.05 mL of sample (from the post-decontamination HgCl_2_ aqueous solution with the pH corrected to 5). The amount of mercury absorbed (*q*) per 1 g of aerogel was calculated according to Equation (3):

(3) $$q=\frac{{(c}_{0}-c_{f})\cdot V}{m}$$

where *q* (mg/g) is the amount of Hg^2+^ absorbed by the hydrogel; *c*_0_ and *c_f_* (mg/L) represent the initial and the final Hg^2+^ concentration, respectively; *m* (*g*) stands for the amount of aerogel employed for each decontamination experiment; and *V* (L) represents the volume of solution used. For the aerogel samples, the reflection spectra were registered using an integrating sphere. The fluorescence/photoluminescence spectra were registered using a Jasco FP-6500 Able Jasco spectrofluorometer.

ICP-MS (Inductively Coupled Plasma) measurements were performed using an ICP-MS 7700s (Agilent Technologies, Santa Clara, CA, USA) device and the following instrumental parameters: plasma gas Ar (6.0 or 99.9999 analytical purity) 15 L/min Ar; carrier gas: 0.7 L/min Ar; makeup gas: 0.5 L/min Ar; nebulizer pump: 0.1 RPS; and collision gas (octopole reaction cell): 4 mL/min He. The mass spectrometer tuning was performed according to the specifications of the manufacturer, with a multielement standard solution—called tuning solution (5 elements in digestion matrix (nitric acid 5% and hydrochloric acid 0.5%): Li, Co, Y, Ce, and Tl; 5 ppb each)—in both “no gas” and “He” modes (in the collision cell—an octopole), calibrating the mass axis in 3 points for small, medium, and high masses, and optimizing the ion optics for the best signal to noise ratio. The data acquisition parameters of the mass spectrometer used were 0.9 s integration time per channel or 2.7 s per *m*/*z* (3 channels out of 20 available were monitored). Monitored masses (natural isotopic abundance, %): ^45^Sc—internal standard abbreviated ISTD (100%), ^89^Y—ISTD (100%), ^200^Hg (23.10%), ^202^Hg (29.86%), and ^209^Bi—ISTD (100%). Calibration standards were acquired to represent the calibration curves for each isotope (5 concentrations per element: 0.0001%, 0.001%, 0.01%, 0.1%, and 1% of the spike 100% concentration (946 ppm)), from the most diluted calibration standard to the most concentrated one. The samples were subsequently measured, with 2 wash blanks between two consecutive samples to prevent “memory effects” (carryover). All the labware used for this study (consisting of perfluoroalkoxy alkane (PFA) parts) was thoroughly cleaned before use, consisting of washing cycles with nitric acid vapors, ultrapure water leaching, a washing cycle with ultrapure water vapors, ultrapure water leaching, rinsing, and final drying at 50 °C. A matrix solution of 5% + 0.5% nitric and hydrochloric acids in water (all ultrapure) was prepared by using ultrapure nitric and hydrochloric acids (Merck, Darmstadt, Germany). Mono-element 1000 mg/L Reference Material Mercury solution (Merck, Germany) was used for the preparation of the spike solutions. Two spike solutions were added to the positive quality control samples (Spike 100%-946 ppm and Spike 10%-94.77 ppm). Sc, Y, and Bi were used separately to prepare an internal standard solution. The accuracy of the method is between 107%, and the acceptance limit is (70–150%). The internal standard used for normalization was ^209^Bi due to the fact that it is not present in the measured samples and has a variation of less than 5%. Two subsamples of 0.04 ± 0.002 g were collected from the testing materials. Each mass was accurately weighed into a PTFE digestion vial and recorded. In order, the following were added to the vial: 0.1 g of MXS, 7.50 g of ultrapure HNO3 60%, and 1.15 g of ultrapure HCl 30%. For the spike experiment, 0.1 g of both solutions (Spike 100%-946 ppm and Spike 10%-94.77 ppm) were added each in two vials together with 7.50 g of ultrapure HNO_3_ 60% and 1.15 g of ultrapure HCl 30% (Table S2). The vials were capped and placed in a 15-position PTFE rack for automatized microwave digestion. The digestion was performed on a Milestone Ultrawave digestor, as follows: the digestion tank was sealed airtight, pressurized with nitrogen (5.0 or 99.999 analytical purity) at 40 bar and subject to a temperature program, with a maximum microwave power of 1500 W. The applied temperature program was as follows: starting from room temperature (~22 °C), ramp to 50 °C in 5 min; ramp to 250 °C in 40 min; and 40 min isothermal at 250 °C. The resulting total digestion time was 85 min. Afterward, the digested solutions were slowly cooled (~40 min) and depressurized at 40 °C until they reached room temperature and pressure conditions. One final dilution was performed with 45 g of ultrapure HCl 0.5 (MSX2), resulting in a final solution mass of ~50 g, which corresponds to a dilution factor of ~1:1300, and a second solution with a total dilution factor of ~1:130,000 with (MSX1). Method quantitation limits are presented in Table S1.

## Figures and Tables

**Table 1:** Comparative presentation of some colorimetric and fluorometric probes in the literature for Hg^2+^.

Molecule	Solvent System	Type	Target	LOD	Ref.
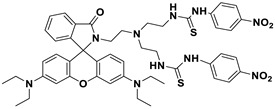	HEPES: MeOH 7:3	Fluorimetric	Hg^2+^	0.11 μM	[56]
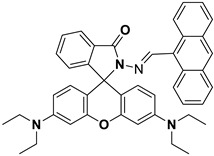	DMF	Fluorimetric	Hg^2+^	0.87 μM	[57]
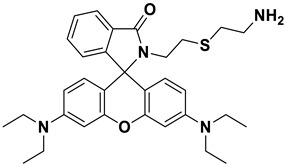	CH_3_CN: HEPES 1:99	Fluorimetric	Hg^2+^	0.14 μM	[58]
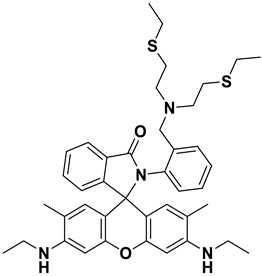	Acetic acid 50 mM pH 5.25	Fluorimetric	Hg^2+^	0.001 nM (0.27μg L^−1^)	[59]
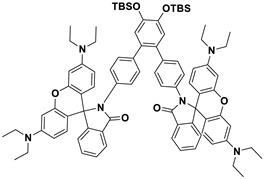	THF	Colorimetric	Hg^2+^	0.1 μM	[60]
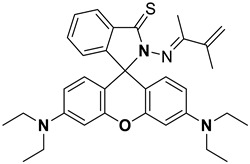	EtOH: HEPES 1:1	Colorimetric	Hg^2^	0.13 μM	
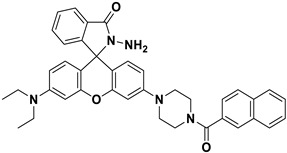	CH_3_CN: H_2_O 7:3	Fluorimetric	Hg^2+^	0.38 μM	[61]
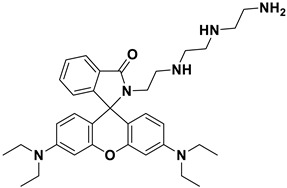	EtOH: H_2_O 1:1 pH 5	Colorimetric	Hg^2+^	2.75 nM (0.55 ppm)	This work

**Table 2:** Hydrogel formulations.

Sample Code	Chelating Agent (g)	RTTA (g)	PVA Solution, 5 wt.%, (g)	AMPSA (g)	NVP (g)	MBA (g)	Photoinitiator, (g)
Bk	-	0.005	10	1.725	3.33	0.1	0.028 g
Phytic acid	0.30	0.005	10	1.725	3.33	0.1	0.028 g
HEXA	0.20	0.005	10	1.725	3.33	0.1	0.028 g
TETRA	0.13	0.005	10	1.725	3.33	0.1	0.028 g
EDTA	0.15	0.005	10	1.725	3.33	0.1	0.028 g

## Data Availability

All data and materials are available on request from the corresponding author. The data are not publicly available due to ongoing researches using a part of the data.

## Supplementary Materials

The following supporting information can be downloaded at: https://www.mdpi.com/article/10.3390/gels10020113/s1, Figure S1—SEM images of the lyophilized hydrogels; Figure S2—FTIR plots; Figure S3—UV-Vis calibration curve; Scheme S1. RTTA Hg^2+^ interaction steps and mechanism for absorption and emission properties modification; Scheme S2. Method principle; Figure S4—Images of RTTA solution color modification due to interaction with different cations; Figure S5—Images of RTTA solution emission modification due interaction with cations; Figure S6—ICP-MS calibration; Table S1. ICP-MS Accuracy; Table S2. Spike Concentrations.
