# Supplementary material for: Dual-Responsive Hydrogels for Mercury Ion Detection and Removal from Wastewater
Source: Gels. 2024 Feb 1;10(2):113. doi: 10.3390/gels10020113 (PMC10887514; doi:10.3390/gels10020113)
Supplement: Supplementary file 1 [file gels-10-00113-s001.zip › gels-2824653-supplementary.pdf]

gels

Supplementary Materials

Dual-Responsive Hydrogels for Mercury Ion Detection and Removal from Wastewater

Aurel Diacon [1,2], Florin Albota [3], Alexandra Mocanu [2,4], Oana Brincoveanu [4,5], Alice Ionela Podaru [1], Traian Rotariu [1], Ahmad A. Ahmad [6], Edina Rusen [2,*] and Gabriela Toader [1,*]

[1] Military Technical Academy "Ferdinand I", 39-49 G. Cosbuc Blvd., 050141 Bucharest, Romania; aurel_diacon@yahoo.com (A.D.); podaru.alice04@gmail.com (A.I.P.); traian.rotariu@mta.ro (T.R.)
[2] Faculty of Chemical Engineering and Biotechnologies, National University of Science and Technology Politechnica Bucharest, 1–7 Gh. Polizu Street, 011061 Bucharest, Romania; alexandra.mocanu@imt.ro
[3] Horia Hulubei National Institute of Physics and Nuclear Engineering, 30 Reactorului Street, 077125 Magurele, Romania; florin.albota@nipne.ro
[4] National Institute for Research and Development in Microtechnologies—IMT Bucharest, 126A Erou Iancu Nicolae Street, 077190 Bucharest, Romania; oana.brincoveanu@imt.ro
[5] Research Institute of the University of Bucharest, University of Bucharest, 90 Sos. Panduri, 050663 Bucharest, Romania
[6] Department of Physical Sciences, Jordan University of Science & Technology, P.O. Box 3030, Irbid 22110, Jordan; sema@just.edu.jo
* Correspondence: nitagabriela.t@gmail.com (G.T.); edina_rusen@yahoo.com (E.R.)

Contents

Figure S1 – SEM images of the lyophilized hydrogels 2
Figure S2 – FTIR plots 5
Figure S3 – UV-Vis calibration curve 5
Scheme S1. RTTA Hg^{2+} interaction steps and mechanism for absorption and emission properties modification 6
Scheme S2. Method principle 6
Figure S4 - Images of RTTA solution color modification due to interaction with different cations 7
Figure S5 - Images of RTTA solution emission modification due interaction with cations 7
Figure S6 – ICP-MS calibration 8
Table S1. ICP-MS Accuracy 8
Table S2. Spike Concentrations 8

Sample

Figure S1 – SEM images of the lyophilized hydrogels

EDTA

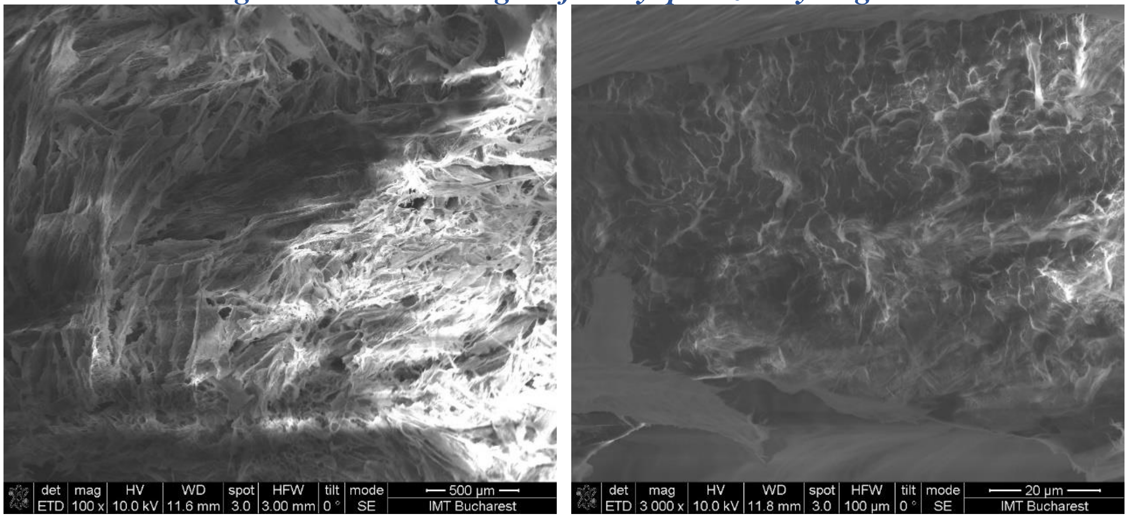

c

TETRA

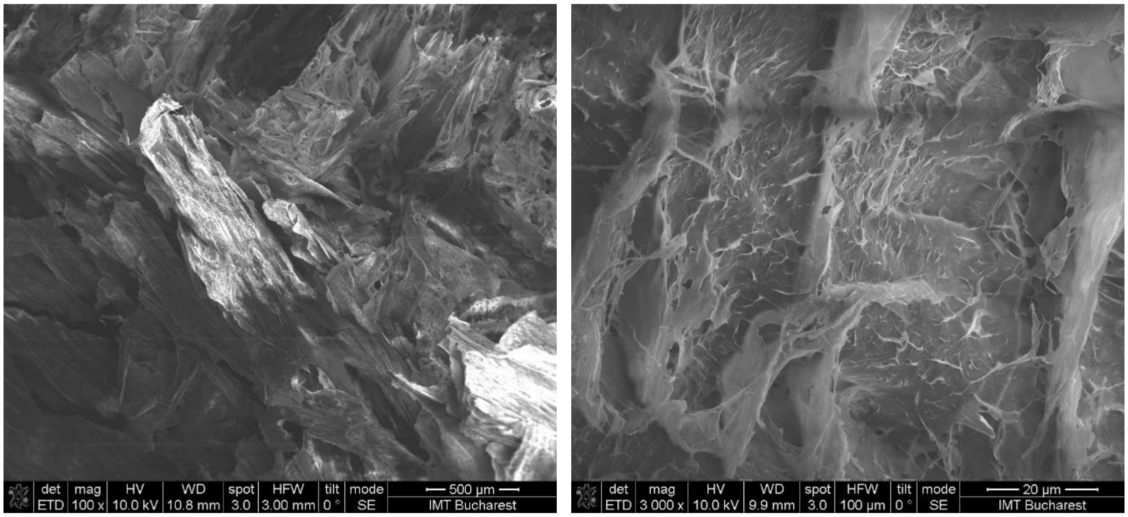

d

HEXA

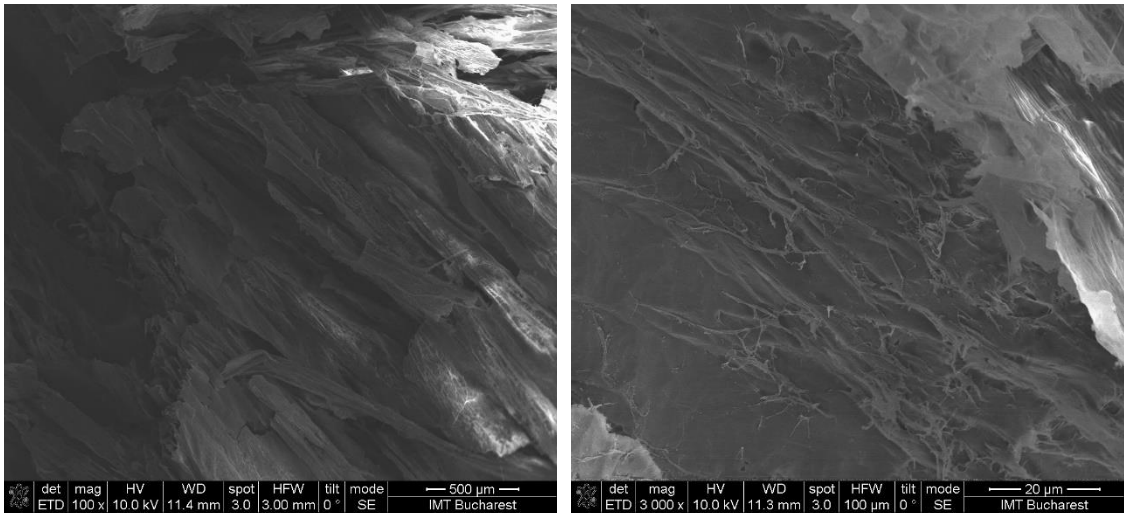

e

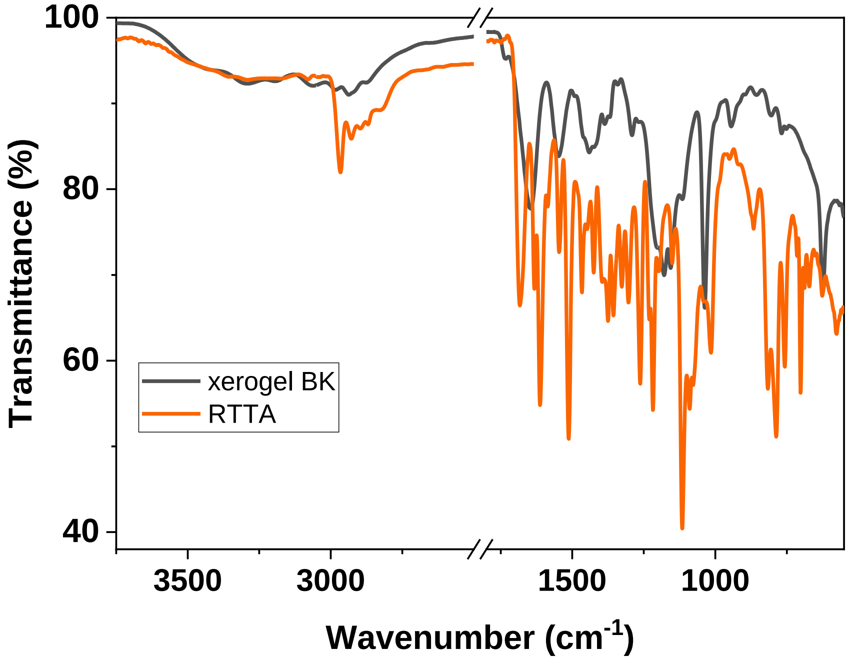
100
80
60
40
Transmittance (%)
xerogel BK
RTTA
3500
3000
1500
1000
Wavenumber (cm^{-1})

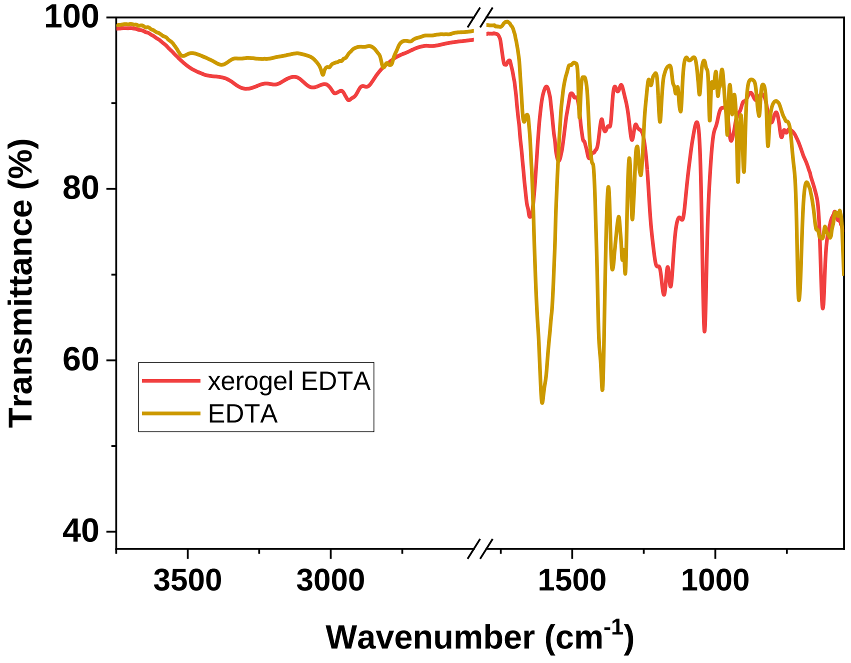
100
80
60
40
Transmittance (%)
xerogel EDTA
EDTA
3500
3000
1500
1000
Wavenumber (cm^{-1})

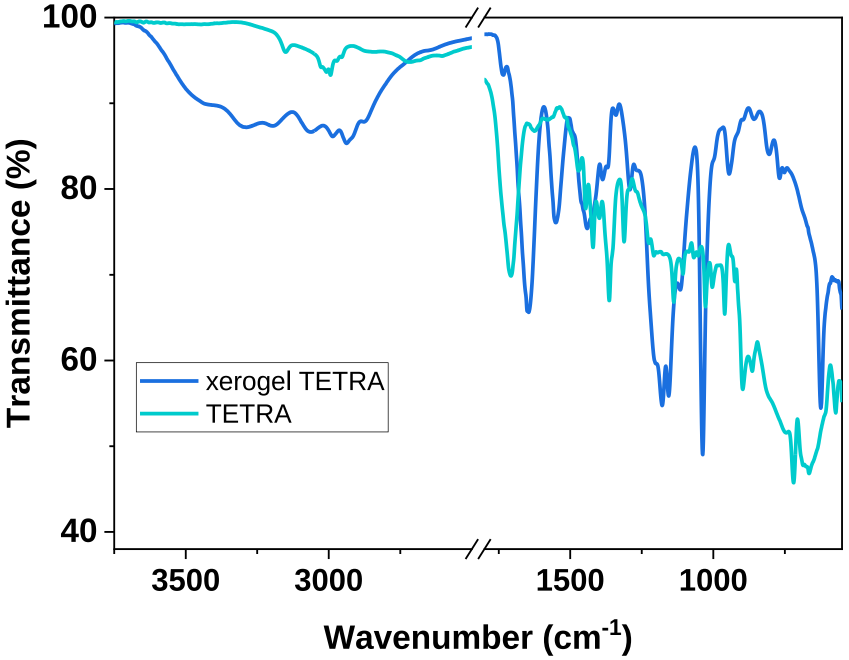
100
80
60
40
Transmittance (%)
xerogel TETRA
TETRA
3500
3000
1500
1000
Wavenumber (cm-1)

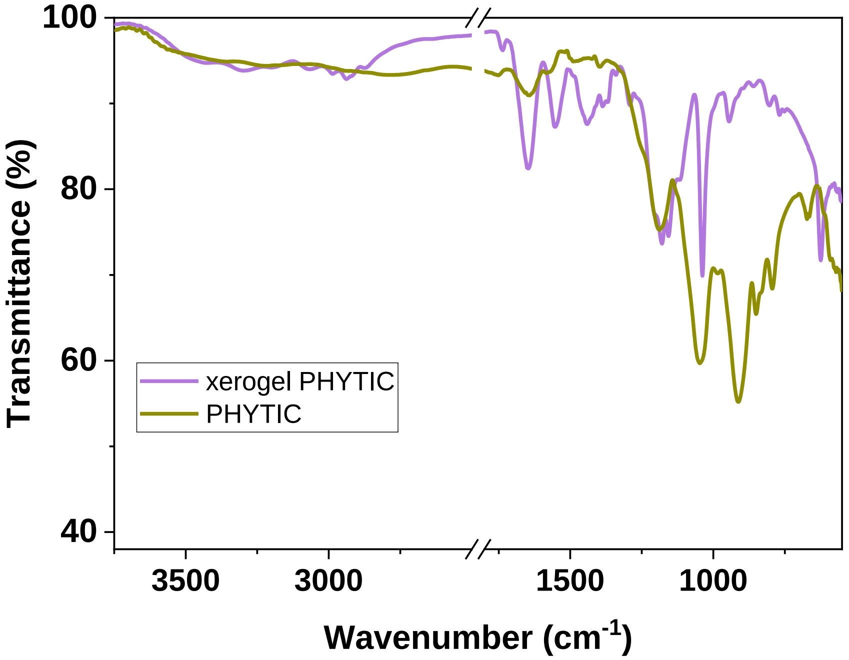
100
80
60
40
Transmittance (%)
xerogel PHYTIC
PHYTIC
3500
3000
1500
1000
Wavenumber (cm-1)

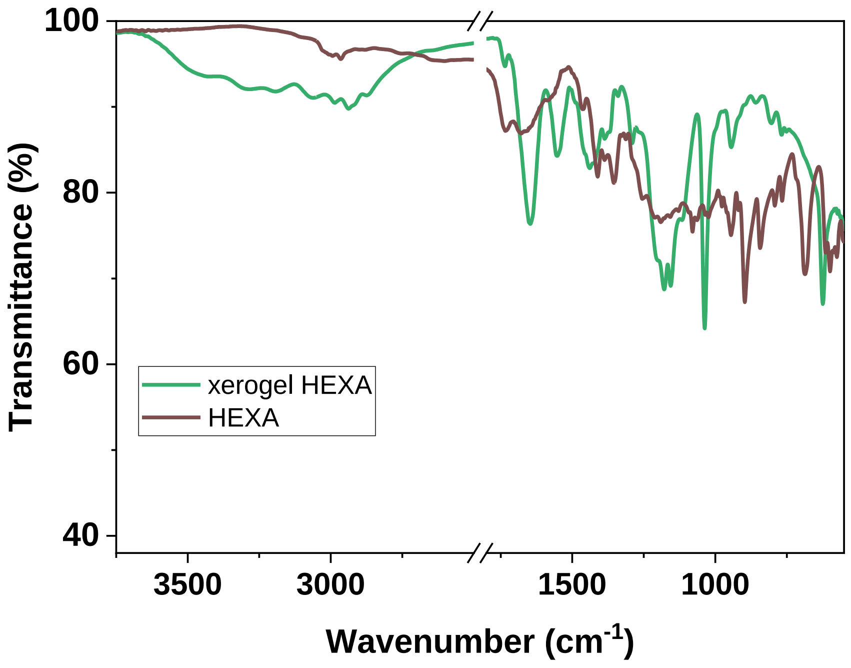

Figure S2 – FTIR plots

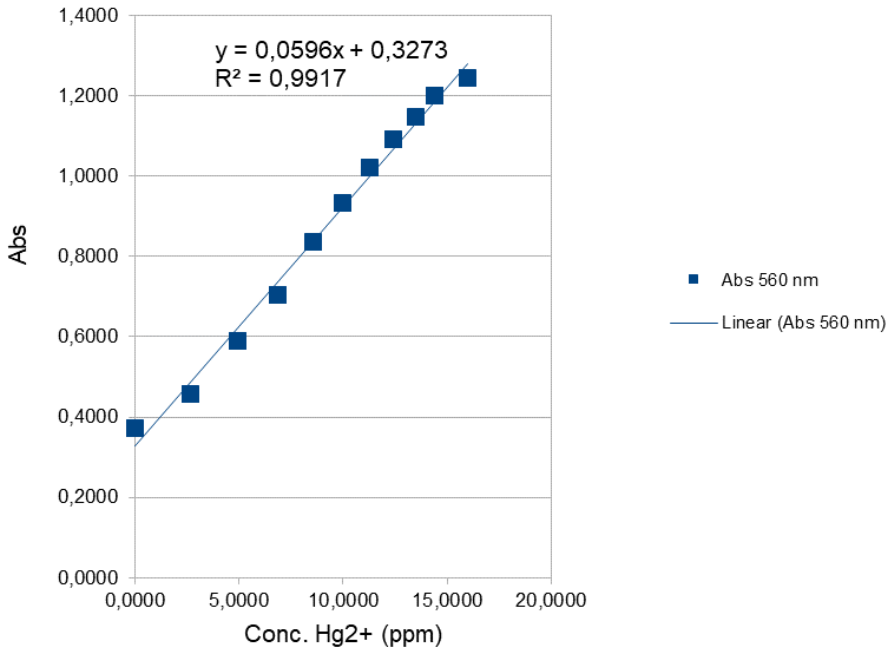

Figure S3 – UV-Vis calibration curve

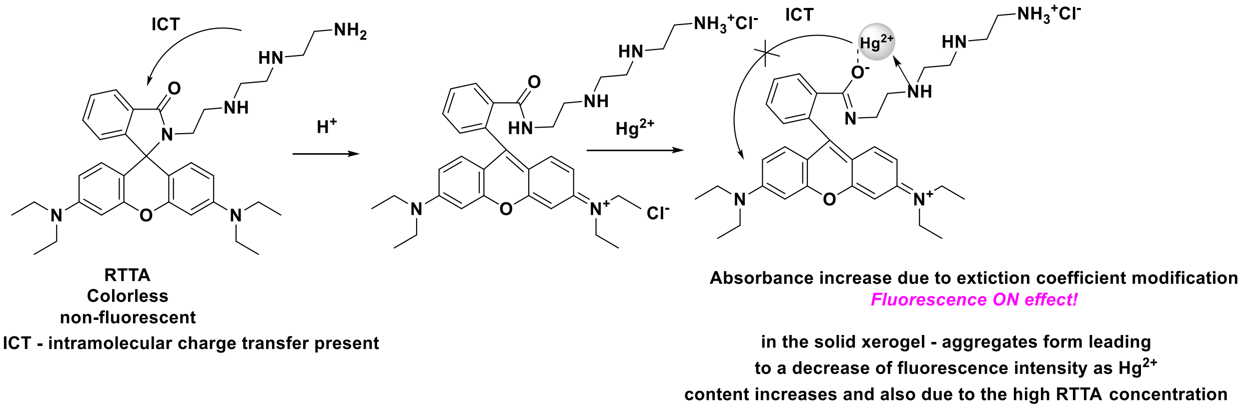

Scheme S1. RTTA Hg^{2+} interaction steps and mechanism for absorption and emission properties modification

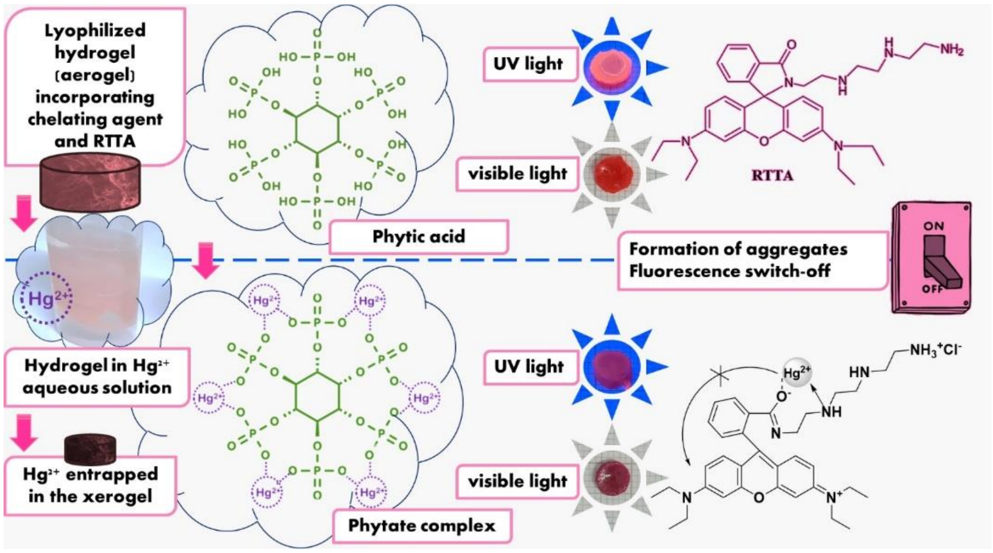

Scheme S2. Method principle

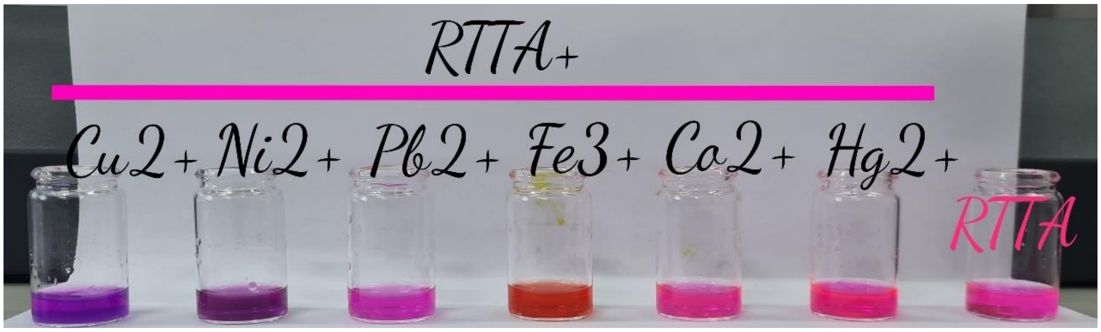

Figure S4 - Images of RTTA solution color modification due to interaction with different cations

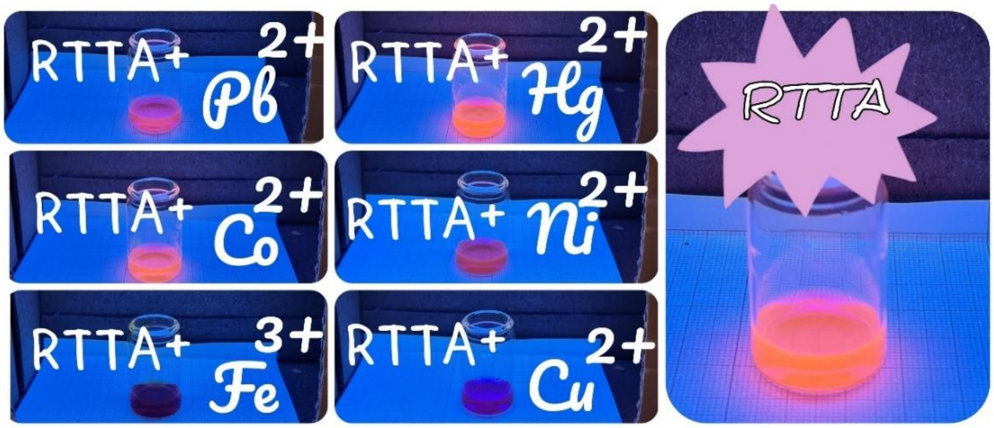

Figure S5 - Images of RTTA solution emission modification due interaction with cations

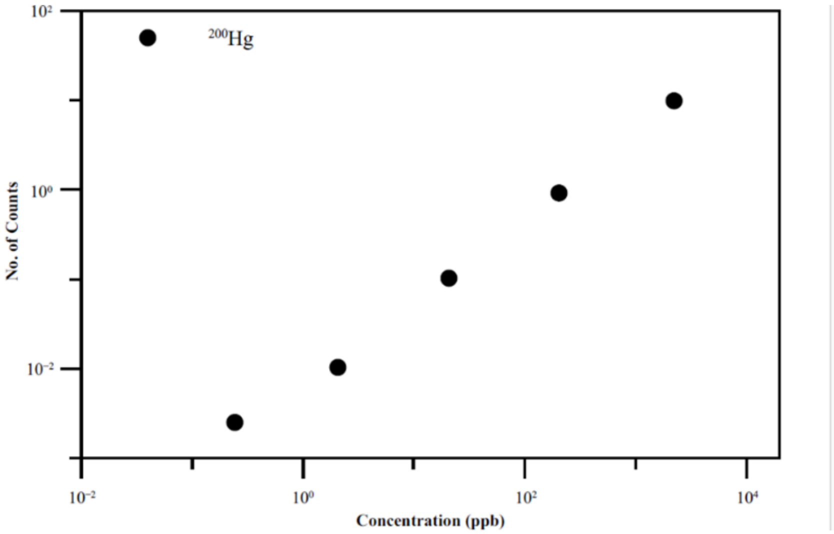

gels

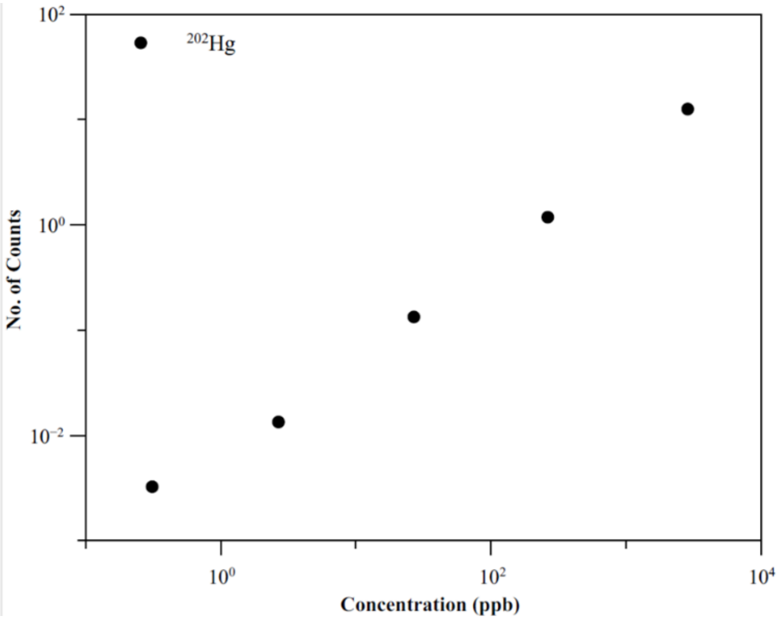

Figure S6 – ICP-MS calibration

Table S1. ICP-MS Accuracy

	^{200}Hg [1]	^{202} Hg [1]
Accuracy	107.07%	107.20%

Table S2. Spike Concentrations

	Spike 100 %	Spike 10%
Concentration (ppm, mg/kg)	946	94.77
